# Challenges of the Health Research System in a Medical Research Institute in Iran: A Qualitative Content Analysis

**DOI:** 10.5539/gjhs.v7n1p69

**Published:** 2014-08-15

**Authors:** Mohammadkarim Bahadori, Khalil Momeni, Ramin Ravangard, Maryam Yaghoubi, Khalil Alimohammadzadeh, Ehsan Teymourzadeh, Ali Mehrabi Tavana

**Affiliations:** 1 Health Management Research Center, Baqiyatallah University of Medical Sciences, Tehran, Iran; 2 School of Management and Medical Information Sciences, Shiraz University of Medical Sciences, Shiraz, Iran; 3 Health Services Management Department, Tehran North Branch, Islamic Azad University, Tehran, Iran

**Keywords:** evaluation, reaserch, qualitative content analysis, medical, health, Iran

## Abstract

**Background and Aim:**

Medical research institute is the main basis for knowledge production through conducting research, and paying attention to the research is one of the most important things in the scientific communities. At present, there is a large gap between knowledge production in Iran compared to that in other countries. This study aimed to identify the challenge of research system in a research institute of medical sciences in Iran.

**Matherials and Methods:**

This was a descriptive and qualitative study conducted in the first 6 months of 2013. A qualitative content analysis was conducted on 16 heads of research centers in a research institute of medical sciences. The required data were gathered using semi-structured interviews. The collected data were analyzed using MAXQDA 10.0 software.

**Results:**

Six themes identified as challenges of research system. The themes included barriers related to the design and development, and approval of research projects, the implementation of research projects, the administrative and managerial issues in the field of research, the personal problems, publishing articles, and guidelines and recommendations.

**Conclusion:**

Based on the results of the present study, the following suggestions can be offered: pushing the research towards solving the problems of society, employing the strong executive and scientific reseach directors in the field of research, providing training courses for researchers on how to write proposals, implementing administrative reforms in the Deputy of Research and Technology, accelerating the approval of the projects through automating the administrative and peer-reviewing processes.

## 1. Background

Medical research institute is the main basis for knowledge production through conducting research (Park & Leydesdorff, 2010; Salas Velasco, 2014). Universities and research institutes are the major institutions which have the prominent role in the research and scientific development through activities such as determining research topics and priorities needed for the community, adoption and implementation of researche, etc.(Hosseinpour, 2011; Kobová, 2014). Research means searching to find facts and knowledge. Also, reaserch literally means searching, probing, and investigating. In other words, this can be seen as a movement leads to the development goals and objectives and ultimately improved quality of life (O’Keeffe, Ganesan, King, & Murphy, 2012; Wilkinson, 2013). A research is a strategy to answer a question or solve a specific problem through collecting, reviewing and analyzing, and interpreting data regularly (Burton & Walters, 2013).

Therfore, paying attention to the research is one of the most important issues in the scientific communities. Undoubtedly, scientific advances are the only support and backing that can guarantee the sustainability and durability of the country’s economic and political independence in the future. In the meantime, the country medical community, which has constantly been diligent and studious in the science of medicine and helping the country to improve the pelople’s health, has greater need for research because of the complexities of health issues and it is hoped to gain its proper and rightful place through creating favorable environments and opportunities and encouraging talented individuals (Dorsey et al., 2013; Nielsen, 2014; Soria, 2013).

It is a very important issue to the extent that has resulted in all industrialized and developing countries increase the volume of their research budgets and investments. Also, developed countries are investing in research to maintain their position or increase their supremacy in the international competition. Accordingly, it can be claimed that there is a direct relationship between conducting research and real progress of every country, and we cannot achieve, in a real sense, sustainable and social development until our country and research institutes do not use the achievements of strategic, applied and development research in their social, economic, cultural and educational planning (Sabzevari, Mohammad Alizadeh, & Aziz Zadeh Foroozi, 2000; Van Helden, 2012).

The results of statistical comparison of the different countries show that research budget is fixed in the world. Developed countries, on average, spend 2% to 3% of their GDP per capita on research while Iran has spent only 0.3% to 0.6% of its GDP on research between 1991 and 2001 (“Reporting of research activities performance,” 1999).

In Iran, a large part of the research capacity has been concentrated in the contry’s universities, and fifty-seven thousand faculty members are there at public and Islamic Azad (open) universities (Kassai, Ahmadi, Liaghat, Aboutorabi, & Kassai, 2013). According to the statistics, the total number of Iranian articles indexed in 2008 was 13,568 articles which showes that, on average, there has been one scientific article published for every four members of the faculty (Sabori, 2008). However, this index has been 40 in each year in the public universities in Thailand (Wichian, Wongwanich, & Bowarnkitiwong, 2009) which shows a great difference compared to that in Iran.

However, at present, there is a large gap between knowledge production in Iran compared to that in other countries, and the statistics show that despite the fifty percent growth in Iran scientific production in 2010, there still is a large gap between Iran and other countries in the region (Sabori, 2010). Therefore, the first step in order to improve the research in the community is to achieve the proper understanding of the capabilities and, also, to realize the strengths and weaknesses of research programs. Identifying the shortcomings and knowing how to achieve the goals of research programs and the rate of this achievement are the basic tools for research decision makers, planners and policy makers to make necessary decisions to achieve goals, improve methods and increase efficiency (Sereshti, Kazemian, & Daris, 2010).

Nevertheless, the results of different studies conducted in Iran show that there are factors considered as major barriers to research. The committee of identifying barriers to research and innovation in Iran after 10 years of reviewing published books, articles, and other sources found seven barriers to research, including management, research policy making, the barriers related to the research culture, the barriers related to researchers, the barriers related to the standard space for research, the barriers related to research terms and regulations, barriers related to research budgets, and barriers to utilize research findings (Hosenipour, 2012). In studies conducted in other countries, researchers have found some other barriers to research, including workplace and demographic characteristics (Carrion, Woods, & Norman, 2004), and the lack of access to information resources (Kotrlik, Bartlett, Higgins, & Williams, 2002). Watty and colleagues (2008) also showed that research had a positive relationship with teaching and a negative relationship with executive positions (Watty, Bellamy, & Morley, 2008).

The present study aimed to identify the challenge of research system in a research institute of medical sciences in Iran.

## 2. Matherials and Methods

### 2.1 Research Design and Setting

This was a qualitative study conducted in a research institute of medical sciences in Iran in the first 6 months of 2013. A qualitative content analysis was conducted on all 16 heads of research centers in a research institute of medical sciences selected by purposeful sampling. These heads were involved in both research activities and administrative and managerial activities and, therefore, were familiar with the challenges in both fields. The required data were gathered using in-depth and semi-structured interviews. All of participants were male and faculty member. All of them aged between 40-50 years old. They had the experience of faculty memebr from 5 to 16 years.

### 2.2 Data Collection

At first, three in-depth interviews were conducted. Then, the related literature, including articles, books, journals and magazines, web pages, etc. was reviewed and the various aspects of the study and the conceptual framework were investigated. After reading them and understanding the various aspects of the research, the subjects of semi-structured interviews were identified. After conducting all interviews, they were transcripted, and then were analyzed using MAXQDA 10.0 software. Next, the structuring problems were identified and the practical solutions were provided.

The transcribed texts were analyzed using qualitative content analysis. This approach emphasizes on the subject and context, differences and similarities within sub-themes and themes. Inductive content analysis had used in different stages. The content of each interview was read to obtain an overall perception of the data and to obtain ideas for next analysis. Then, all the texts were divided into meaning units and the meaning units were condensed into open coding. After that, open coding had developed and sub-themes and themes were created (23-24).

The main research questionwas: “Can you explain about the research challenges of your research institute?”

### 2.3 Ethical Consideration

The study was approved by the Medical Research Ethics Committee of the Baqiyatallah University of Medical Sciences. Before conducting interviews, the respondents were given an information sheet consisting of all the required information about the research and their participation in this study. They were given enough time to answer the questions. In addition, the verbal consent was obtained from all respondents to participate in this study and all of them were assured of the confidentiality of their responses.

### 2.4 Data Analysis

Credibility of the data was done using member checking. The finding of the analysis was given to the participants in order to obtain assurance that the researchers were presenting their ideal worlds. In addition, faculty members did member checking of the transcripts, subcategories, and themes (Bahadori, Ibrahimipour, & Farzaneh, 2012; Mahmoodishan, Alhani, Ahmadi, & Kazemnejad, 2010).

## 3. Results

Overal, 16 heads of research centers of the research institute of medical sciences were interviewed. After transcripting interviews conducted and analyzing them, 6 themes and 31 sub-themes were identified. Below, after expressing each of the themes and their sub-themes, some of the interviewees’ quatations have been given. Six themes and 31 sub-themes were identified as follows:

### 3.1 Theme 1: The Design and Development, and Approval of Research Projects

Eight sub-themes were identified in this theme, including inappropriate choice of research topics, improper development of research proposals, the lack of an integrated system related to the research topics, the administrative bureaucracies and lengthy approval process of research projects, using relationships and associations instead of the rules in approval of research projects due to the high academic degrees of their supervisors, problems with peer-reviewing the research projects, the lack of research centers’ independence in approval of research projects, and the lack of a clear plan to design and develop the research projects. Eitht sub-themes were identified as follows:

- Inappropriate Choice of Research Topics
  “The most important issue is determining the research topic. Usually, we are having trouble with determining the topic which apparently is based on community needs, however in practice, this is not so. We have not recognized the needs well and this is the origin of the problem at all stages of the research, from preparing an article to its publication as well as the applicability of research projct (M8). Choosing a topic here is only because of a thing to be done and a report to be prepared, and it is our duty to teach people and individuals how to determine the topic of research projrct which, in itself, is an art and they should know how to find ideas” (M5).
- Improper Development of Research Proposals
  “Writing a proposal in each center is somewhat differe from that in other centers due to its specialized nature. This issue should also be considered. Many of our researchers do not know writing proposals well”(M5).
- The Lack of an Integrated System Related to the Research Topics
  “Some of research topics are repetetive and duplicated, and there is not any integrated system available online to the researchers guiding appropriately them to avoid the duplication of research activities” (M 1,8,11).
- The Administrative Bureaucracies and Lengthy Approval Process of Research Projects
  “The projects are approved in the center and are sent to the Deputy of Research for comments and revisions, and this takes a long time a research project to be carried out after its approval” (M1). Also, because the approval process is done through a paper system, this process will take a long time. In addition, given that only the heads of centers have access to the automation, this also makes the process longer” (M3).
- Using Relationships and Associations instead of the Rules in Approval of Research Projects due to the High Academic Degrees of their supervisors
  “Usually in approving the research projects, referees and peer reviewers pay attention to the academic degrees of their supervisors instead of the content of project and that which problem in the field of health can be solved by conducting that research project (M6), and those projects which have more famous supervisers and have supervisors with higher academic degrees will have priority to be approved, and this would not be an appropriate process for young researchers who are highly motivated to research”(M11).
- Problems with Peer-Reviewing the Research Projects
  “Unfortunately, in projects peer-reviewing we are faced with the problem that the related referees and reviewers ususlly don’t review and study the projects before coming to the arbitration hearing and comment on them during that short session, which is not appropriate” (M6,7).
- The Lack of Research Centers’ Independence in Approval of Research Projects
  “The research prejects should be approved by the University Deputy of Research again after being approved by the research centers, which is duplication and causes the slowness in implementing the project. The research centers themselves should be independent and have the autonomy for final approval of the projects” (M8,9).
- The Lack of a Clear Plan to Design and Develop the Research Projects
  “The main problem in designing and developing research projects is that there isn’t a clear plan and a clear roadmap based on facts in the research institute” (M2). “The research institute does not have any clear path for research, and if has, it is unclear and ambiguous to researchers, especially in the basic sciences” (M9).

### 3.2 Theme 2: The Implementation of Research Projects

The following five sub-themes were identified in this theme: the lack of funds and budgets for research projects, poor control and supervision over the implementation of the research project, the lack of timely financial support during the project implementation, inadequate cooperation of research centers in data collection, delays in the implementation of research projects and the lack of project supervisors’ cooperation. Five sub-themes were identified as follows:

- The Lack of Funds and Budgets for Research Projects
  “The policies of funding and budgeting are not appropriate because the budgets are traditionally set every year and there is not any specific plan for spending budget lines” (M1,7). “These funds and budgets are allocated routinely and it is not monitored.” (M4).
- Poor Control and Supervision over the Implementation of the Research Project
  “There is no proper control and supervision over the implementation of the research project and whether the project has finished accurately and in the predetermined time” (M3).
  “Monitoring of research projects is similar to the work of a construction supervisor engineer which signs and approves the project without exercising any control and supervision. While monthly progress report should be provided for the project and this report should be prepared according to a certain format, and the reasons for the slowness of the project, if any, should be given” (M5).
- The Lack of Timely Financial Support during the Project Implementation
  “Given the economic and political situation of the country, the cost of projects may increase. This should be considered in budgets so that the increase in project costs does not interrupt the implementation of the project” (M4).
- Inadequate Cooperation of Research Centers in Data Collection
  “Collecting data in research centers is very difficult due to their nature. The heads and personnel of these centers don’t cooperate with researchers fully mostly due to the lack of adequate knowledge and attitudes towards these projects” (M3,5,6,8,10). “We inevitably have to accept some problems in data collection. For example, due to the hospital high workload and lack of sufficient time for staff to complete the questionnaires, the researcher should consider a right and good time to collect the required data, and even a special budget should be taken into account for those individuals who are involved in the project, and in addition to the spiritual dimension, the material and financial dimension should also be supported” (M4).
- Delays in the Implementation of Research Projects and the Lack of Project Supervisors’ Cooperation
  Some projects aren’t tracked after their approvals and because there isn’t sufficient monitoring, the project isn’t implemented for a while. Even the project supervisor, due to his/her problems, doesn’t desire to replace himself/herself with other person or persons participated in the project to implement it because he/she further considers the financial dimension of the project” (M2,4,9).

### 3.3 Theme 3: The Administrative and Managerial Issues in the Field of Research

Eight sub-themes were identified in the administrative and managerial issues in the field of research, including failure to timely provision of the required equipment for implementing the research project, the lack of research methodology consultant, the lack of educated and skilled researchers, the lack of adequate supervision of the managers over research funds and budgets, the lack of accurate evaluation of the research activities of researchers and faculty members, not using the research findings in making decisions, the lack of clear long-term objectives of research centers, the lack of specialized and skilled research directors in research centers. Eitht sub-themes were identified as follows:

- Failure to Timely Provision of the Required Equipment for Implementing the Research Project
  “In terms of equipment and medicines required, we are in a bad situation and given that the country is sanctioned (is dependent on overseas to provide its required equipment), failure to timely provision of the required equipment can result in the slowness in implementing the project. Therefore, the required equipment should be provided in accordance with the project and in a timely manner” (M5,10).
- The Lack of Research Methodology Consultant
  “A statistician should attend in all research sessions to determine how the research results have been achieved and whether the numbers and figures are right or not because the main part of a research project is its statistical discussion” (M1). “All research proposals should have a statistician and an epidemiologist as its consultatnts. This is done, but not always. Of course the consultation should be provided by a coordinated group of skilled and expert consultants or by a methodologist”(M2).
- The Lack of Educated and Skilled Researchers
  “Individuals who are skilled in research play an important role in reaching research objectives. In research centers, the number of qualified and expert personnel in research who are involved in research seriously is not sufficient” (M2,7).
- The Lack of Adequate Supervision of the Managers over Research Funds and Budgets
  “The supervision on research budgets is not sufficient to monitor whether they are spent correctly or not and whether the budget allocated to a project is appropriate or not. We should have feedback on the budgets dedicated to the projects” (M1,3,5). To implement research, the funds and budgets should be supervised adequately, and everyone doing anything should be accountable for that. Projects that are supposed to be done should be based on community needs and demands. After completion of the project and presenting its findings to the center that has required and requested it, we need to ask whether your problem has been solved or not. If its answer is positive, then the research has well been done” (M4,7,10).
- The Lack of Accurate Evaluation of the Research Activities of Researchers and Faculty Members
  “There isn’t any careful evaluation of the research activities of researchers and faculty members, and indicators considered to assess researchers from every aspect are more related to the number of published articles” (M1,11).
- Not Using the Research Findings in Making Decisions
  “What is the aim of conducting research? To answer a question or meet a need. We should consider that this research approach is to solving a problem, or more to publishing articles” (M1,3,4). “It should be noted that the research conducted should be applied, otherwise it is a waste of money, and we should not frustrate our human resources through conducting inapplied and theoritical research because we need them and we should constantly practice to implement the projects that are applied” (M6).
- The Lack of Clear Long-Term Goals of Research Centers
  The problem research centers are faced with is the lack of clear long-term objectives; overall, there are not any long-term and short-term objectives, and even this problem also exists in the Ministry. For example, there are about 20 molecular biology centers in Iran, but they all only work in the field of molecular diagnosis and all are intertwined. The Ministry of Health should require each of them to work in a specialized field; for example, the research center of Bushehr University should work on oil pollution, the other center on aquatic organisms, etc. (M10).
- The Lack of Specialized and Skilled Research Directors in Research Centers
  “The research directors are not highly professional and specialized, and should have their own specific criteria. A person should be the director of a research center who is a faculty member and has been involved in several research projects as an executive or a co-researcher, as well as should be familiar with the research problems” (M6). “The most of directors of research centers have been selected as research centers’ directors due to lack of manpower (M15).

### 3.4 Theme 4: The Personal Problems

The following three sub-themes were identified in the theme of personal problems: the researchers’ lack of time, financial and economic problems, and the lack of fair and equitable sabbaticals among the staff of research centers. Three sub-themes were identified as follows:

- The Researchers’ Lack of Time
  “Because researchers are engaged in other activities such as teaching and executive activities in addition to research, they are too busy and don’t have enough time” (M2,3,4,13).
- Financial and Economic Problems
  “When a researcher sees that conducting and implementing research doesn’t have any financial and spiritual benefits for him/her, no longer works in the field of research and pays no attention to that and, therefore, they involve in other fields except for research. For example, a physician can earn more income in his/her clinic and sees that research is of no interest to him/her. While we need strong individuals and researchers in the field of research and this field should be paid great attention (M5,6,10,11).
- The Lack of Fair and Equitable Sabbaticals among the Staff of Research Centers
  “In giving sabbatical, also, relationships and associations take priority over the rules, and those who have higher academic degrees are given higher priority to take sabaticals. However, appropriate indicators should be considered for giving sabbaticals” (M16).

### 3.5 Theme 5: Publishing Articles

Two sub-themes were identified in the theme of publishing articles, including the possiblity of not publishing some military articles and, therefore, not receiving the required privillages, and prolonging the process of peer-reviewing and publishing articles. Two sub-themes were identified as follows:

- The Possiblity of Not Publishing Some Military Articles and, Therefore, Not Receiving the Required Privillages
  “Some scientific journals don’t publish the military articles for numerous reasons such as the security of their information. Therfore, these articles are published more in the military scientific journals” (M1,3,8,11).
- Prolonging the Process of Peer-Reviewing and Publishing Articles
  “Some journals peer-review the articles very quickly and the others review them later mostly because of the high number of articles submitted to them. Usually prestigious scientific journals peer-review and publish articles later because of their accurate and detailed assessments” (M2,9).

### 3.6 Theme 6: Guidelines and Recommendations

The following five sub-themes were identified in the theme of guidelines and recommendations: providing training and advice on how to select the research topic and motivate the researchers, providing training courses on research methodology and writing research proposals, developing a suitable process of selecting researchers for taking sabbaticals, reducing bureaucracy in the administrative system of research, and considering a right and good time for data collection. Five sub-themes were identified as follows:

- Providing Training and Advice on How to Select the Research Topic and Motivate the Researchers
  “It is our responsibility to train our researchers in how to select the research topic, and knowing how to determine the topic itself is an art, as well as the researchers should know how to find ideas” (M5). “The other thing that should be considered about the topic is the researcher’s interest in the research topic. Usually we see students conducting and implementing research, while they have no interest in it and when there is no interest, there is, also, no incentive to implement the project. So our duty is to train our researchers in how to select a research topic and motivate them, which should be done efficiently” (M8).
- Providing Training Courses on Research Methodology and Writing Research Proposals
  “It is necessary for professors and faculty members, as the consumers of research findings, to be trained. Because as long as the spirit of research is not widely established among them, the research market will not be expanded enough” (M3).
- Developing a Suitable Process of Selecting Researchers for Taking Sabbaticals
  “One of the innovative ways to strengthen incentives to conduct research, and therefore, to develop research is giving sabbaticals to those researchers who have proved their competence and interest in research. This experience has been employed successfully in other countries, including Japan” (M8).
- Reducing Bureaucracy in the Administrative System of Research
  “Given the importance of administrative system of research, it is required to take necessary measures to make fundamental changes in the administrative and financial regulations governing research system in order to remove inappropriate bureaucratic structure, increase the flexibility in regulations, determine a scientific system for selecting skilled managers, reduce administrative red tape, stabilize management, establish trust to promote research management, and change the available structure, organization, management and planning toward research. Making such changes will provide opportunities to encourage researchers and increase their participation in research” (M1,3).
- Considering a Right and Good Time for Data Collection
  “We inevitably have to accept some problems in data collection. For example, due to the hospital high workload and lack of sufficient time for staff to complete the questionnaires, the researcher should consider a right and good time to collect the required data, and even a special budget should be taken into account for those individuals who are involved in the project” (M4).

## 4. Discussion

The results of the present study showed that overall, the barriers to the research in the studied research institute were due to the problems with the design and development, and approval of research projects, their implementation, the administrative and managerial issues in the field of research, the personal problems, and publishing articles.

Failure to timely provision of the required equipment for implementing research projects, the lack of research methodology consultant, the lack of educated and skilled researchers, the lack of adequate supervision of the managers over research funds and budgets, the lack of accurate evaluation of the research activities of researchers and faculty members, not using the research findings in making decisions, the lack of clear long-term objectives of research centers, the lack of specialized and skilled research directors in research centers were the organizational factors expressed and emphesized by the heads of research centers interviewed.

Sohrabi and Farajollahi (2009) in their study concluded that some problems such as insufficient funds and budgets, time-consuming barriers in collecting data, insufficient directors’ understanding of the research problems, and the lengthy administrative and executive processes were the barriers to research that are consistent with the results of the current study. Sereshti and colleagues (2007), also, in their study foun that the lack of time and being busy, the lack of facilities and equipment, unavailability of and inaccessibility to the required consultants for guidance and consultation, the lack of incentive to research, and not having sufficient privilleges to conduct and implement a research were the main barriers to research. In addition, the results of Sabzevari and colleagues’ study (2000) showed that the organizational barriers to research included the lack of facilities and equipment, and administrative red tape in conducting and implementing the research projects.

Besides the mentioned organizational barriers, the lack of researchers’ time, financial and economic problems, and not equitably distributed sabbaticals among the staff of research centers were, also, the personal barriers.

Dyer and Stren (1990) in their study found the group pressure, the lack of time, the lack of the required resources, the lack of group cooperation, the lack of giving feedback, the lack of collaborative research with other organizations as the organizational barriers to research, as well as the lack of experience, the lack of incentives, and excessive specialization hindering the development of comprehensive views and new approaches as the personal barriers to research which in most cases, their results are similar to the present study results. Bostrom and colleagues (2008), also, in their study concluded that insufficient resources, the lack of enough time, and the unaccessability to reseach articles and reports were barriesr to research.

Furthermore, the lack of funds and bugets, the lack of financial incentives and the lack of motivational factors were the personal and organizational factors expressed by studied interviewees. In addition, in the most of developing countries, insufficient funds and budgets allocated to research, financial and economic problems, rapid changes of managers, and doing things according to taste were among the factors that made it difficult to rely on government funds and budgets. Bureaucracy had also delayed the getting funs and budgets to the researcher so that he/she might ultimately be deterred from conducting and implementing the research (Dorsey et al., 2013). This study results are consistent with the results of the present study.

The lack of specific research needs and priorities of health care system in the studied research institute, the lack of knowledge of experts about how to implement the research project, and the lack of continuous supervision and control over implementation of research projects were some of the sub-themes of design and development, and approval of research projects. Tress, Tress and Fry (2007) in their study showed that the most important barriers were problems with developing communication and collaboration, the lack of time and financial resources in implementing the project, the lack of interest, failure to specify interventions, and the lack of knowledge.

Koohpaie and colleagues (2009) in their study found that the mean intensity differences of research problems among the areas of the design and development, and approval of research projects, the implementation of research projects, the administrative and managerial issues in the field of research, and the personal problems were statitically significant (P < 0.001).

The lack of a useful database in the research institute in the area of design and development, and approval of research projects, the lack of funds and budgets in the area of implementation of research projects, the lack of knowledge of the accounting staff about the process of performing related activities in the area of administrative and managerial issues in the field of research, and the lack of sufficient incentives for research in the area of personal problems had the highest intensity which after the intervention their intensity had significantly reduced (Koohpaie, Yosefi, Komeili Movahed, Ahmari Tehran, & Darabi, 2009).

According to the results of the present study and other studies, there are so many barriers in the different stages of conducting and implementing a research which should be identified and prioritized based on their importance for resolving. Therefore, in the first step the factors should be identified and the needs assessment in research should be carried out.

In a study conducted among the faculty members of the Nursing School in Sydney to detemine effective factors in promoting research, the results ahowed that the first step in improving the research quality was making the needs assessment and analyzing the research needs of faculty members (Gething & Leelarthaepin, 2000). Also, determining the priorities is an important process of managing research in the health sector whose importance is more intuitive, especially in times of resource allocation.

## 5. Conclusion

In summary, the findings of the present study showed that there were so many barriers in the different stages of conducting and implementing research. These barriers were barriers related to the design and development, approval of research projects, the implementation of research projects, the administrative and managerial issues in the field of research, the personal problems, publishing articles, and guidelines and recommendations. Based on the results of the present study, the following suggestions can be offered: enhancing the quality of researchers, pushing the research towards solving the problems of society, providing spiritual and financial supports for the publication of articles in international journals, employing the strong executive and scientific reseach directors in the field of research, providing training courses for researchers on how to write proposals, implementing administrative reforms in the Deputy of Research and Technology, strengthening the research culture in the research system of the research institute, allocating appropriate funds and budgets to the research projects, accelerating the approval of the projects through automating the administrative and peer-reviewing processes, providing standard space, facilities and equipment for research, and providing advanced equipment and equipped laboratories for research.
